# Five Species of *Taxus* Karyotype Based on Oligo-FISH for 5S rDNA and (AG_3_T_3_)_3_

**DOI:** 10.3390/genes13122209

**Published:** 2022-11-25

**Authors:** Zhoujian He, Xiaomei Luo, Yuting Lei, Wei Zhang

**Affiliations:** Southwest Engineering Technology Research Center of Taxus of State Forestry and Grassland Administration, College of Forestry, Sichuan Agricultural University, Huimin Road 211, Chengdu 611130, China

**Keywords:** Oligo-FISH, *Taxus*, physical map

## Abstract

As a relict plant, *Taxus* is used in a variety of medicinal ingredients, for instance to treat a variety of cancers. *Taxus* plants are difficult to distinguish from one another due to their similar morphology; indeed, some species of *Taxus* cytogenetic data still are unclear. Oligo-FISH can rapidly and efficiently provide insight into the genetic composition and karyotype. This is important for understanding the organization and evolution of chromosomes in *Taxus* species. We analysed five *Taxus* species using two oligonucleotide probes. (AG_3_T_3_)_3_ signals were distributed at the chromosome ends and the centromere of five species of *Taxus*. The 5S rDNA signal was displayed on two chromosomes of five species of *Taxus*. In addition to *Taxus wallichiana* var. *mairei*, 5S rDNA signals were found proximal in the remaining four species, which signals a difference in its location. The karyotype formula of *Taxus wallichiana* was 2n = 2x = 24m, its karyotype asymmetry index was 55.56%, and its arm ratio was 3.0087. *Taxus × media*’s karyotype formula was 2n = 2x = 24m, its karyotype asymmetry index was 55.09%, and its arm ratio was 3.4198. The karyotype formula of *Taxus yunnanensis* was 2n = 2x = 24m, its karyotype asymmetry index was 55.56%, and its arm ratio was 2.6402. The karyotype formula of *Taxus cuspidate* was 2n = 2x = 24m, its karyotype asymmetry index was 54.67%, its arm ratio was 3.0135, and two chromosomes exhibited the 5S rDNA signal. The karyotype formula of *T*. *wallichiana* var. *mairei* was 2n= 2x = 22m + 2sm, its karyotype asymmetry index was 54.33%, and its arm ratio was 2.8716. Our results provide the karyotype analysis and physical genetic map of five species of *Taxus*, which contributes to providing molecular cytogenetics data for *Taxus.*

## 1. Introduction

*Taxus* L. is an ancient tertiary relict plant [1] rich in substances such as biflavones, cinnamic acid derivatives, lignans, and steroids [2,3]. In particular, it is rich in the natural taxane diterpene paclitaxel, which, along with some semisynthetic drugs, is used to treat ovarian, breast, skin, and non-small cell lung cancer [4,5]. For the protection of wild *Taxus* plants, the Convention on International Trade in Endangered Species (CITES) has listed all Taxus species in Appendix II. Subsequently, all *Taxus* species in China were included in the National List of Key Protected Wild Plants (http://www.Gov.cn/Baogong/content/2000/content_60072.htm (accessed on 2 September 2022)). Therefore, it is of great significance to study *Taxus*. 

*Taxus* is an important part of Taxaceae, distributed in the northern hemisphere such as in Europe, North America, and East Asia, and it is suitable for growing under humid temperate or tropical mountain forests [1,6,7,8,9]. There are four species and one variety in China, distributed in the northeast, southwest, and south regions [10]. *Taxus* classification is mainly based on morphological description and geographical distribution [11], but some species of *Taxus* have little difference in phenotypic characteristics [9], causing the controversial classification of species of *Taxus*. Its phylogeny remains controversial [7]; some researchers have divided *Taxus* into 7 [12], 8 [13], 9 [14], 10 [10], 10 species and their varieties [11] and 11 [15] species. *Taxus* is classified only by appearance, which is limited and therefore makes it easy to cause classification difficulties. We are looking forward to a more reasonable method. At present, the nuclear and chloroplast genomes of *Taxus wallichiana* var. *mairei* have been sequenced [16] and the chloroplast (cp) genome of *Taxus chinensis* has been sequenced [16]. The mitochondrial (mt) genome of *Taxus cuspidate* has also been sequenced [17]. *Taxus* as a non-model gymnosperm has been studied for organ transcriptome sequencing at present [18]. A draft genome assembly constructed by the whole genome sequencing of *Taxus baccata* has been published [19]. Although some molecular studies have been conducted on certain *Taxus* species [20,21], the whole genome sequencing of *Taxus* is still missing [22]. One of the ways in which plant genomic diversity is expressed is through a wide range of chromosome numbers [23]. However, there are still significant gaps in the overall study of the major cytogenetic characteristics of the Taxaceae genome [22]. Compared with other traits, chromosomal traits are stably informative in plants [24]. Karyotypes mainly contain chromosome numbers and morphology, which reveal cytological characteristics [25]. It is better to understand the organization and evolution of chromosomes in related species [26].

Fluorescence in situ hybridization (FISH) is an important tool for plant karyotypes. which uses oligonucleotide probes [27]. FISH also frequently uses oligonucleotide DNA and other DNA sequences as probes, and has been used on a variety of plants, such as *Pinus densiflora* [28], *Chimonanthus campanulatus* [29], *Citrus clementina* [30,31,32], and *Pinus* [33,34,35]. Telomere repeats (CCCTAAA)_n_, (TTTAGGG)_n_, (TTAGGG)_n_, and (AGGGTTT)_n_ are found in most angiosperms and are located at the end of chromosomes in gymnosperms. *Picea asperata* and *Larix gmelinii* show only terminal sites, but in *Picea sylvestris* [36,37] and *Picea elliottii*, several insertion sites at the centromere sites were also observed [38]. The 5S rDNA and (AG_3_T_3_)_3_ probe combination has been applied to *Hibiscus mutabilis* [39], *Hippophae rhamnoides* [40], and *C. campanulatus* [29]. There is no systematic karyotype analysis of *Taxus*, and the results of previous karyotype studies often have some differences because of the different methods used. To study the karyotypes of five *Taxus* species, we used two oligonucleotide probes that have not yet been used. Our results help to distinguish five *Taxus* species based on chromosome information, and these results will be helpful for the construction of oligonucleotide barcodes in the future.

## 2. Materials and Methods

### 2.1. Seed Materials and Root Tips

The seedlings of *Taxus yunnanensis*, *Taxus × media,* and *T. wallichiana* var. *mairei*. were collected in Caoba town, Yucheng district, Ya’an City, Sichuan province. *Taxus wallichiana* was collected from Xichang city, Sichuan province. *Taxus cuspidate* was collected in Suqian city, Jiangsu province. All five cultivated *Taxus* species are shown in Table 1. For each *Taxus* species, there are three healthy seedlings, each with seeds from the same plant for consistent genotype. The *Taxus* seedlings had been growing for two years before the experiment. The seedling morphology and root tip growth and division of each *Taxus* species were similar. These seedlings were placed at room temperature under natural light conditions in the Chengdu Academy of Agriculture and Forestry Sciences. Approximately 1.5–2.0 cm root tips were treated with nitrous oxide for 4.5 h. After treatment, the root tips were placed in glacial acetic acid for 5 min and then maintained in 75% ethyl alcohol at −20 °C.

### 2.2. Chromosome Preparation

The root tip meristems were washed by using ddH_2_O, and then dispersed with cellulose and pectinase (2:1) at 37 °C for 45 min. After these treatments, the enzyme mixture on the meristems was washed off using ddH_2_O twice, then ethyl alcohol twice. Then, we removed all ethyl alcohol and added up to 20 µL of glacial acetic acid to prepare a suspension. Finally, 10 µL of the mixture was dropped on one slide. We used an Olympus CX23 microscope (Olympus Corporation, Tokyo, Japan) to examine the slides. 

### 2.3. Probe Preparation

The (AG_3_T_3_)_3_ repeat sequence [24] and the ribosome 5S rDNA [41] were used in this study. These two probes were tested for the first time in Taxaceae and these oligonucleotide sequences were produced by Sangon Biotechnology Co., Ltd. (Shanghai, China). The 5′ ends of the probes were labelled with 6-carboxyfluorescein (FAM) or 6-carboxytetramethylrhodamine (TAMRA). The probes were dissolved in 1 × Tris-ethylene diamine tetra acetic acid (TE) and maintained at a concentration of 10 µM at −20 °C.

### 2.4. FISH Hybridization

The slides were fixed in 4% paraformaldehyde for 10 min and oscillated twice in 2 × saline sodium citrate (SSC) buffer for 5 min. After, the samples were dipped in 75%, 95%, and 100% ethyl alcohol successively for 5 min. A total of 60 µL of 70% deionized formamide (FA) was dropped onto the slides and air dried, and coverslips (24 cm × 50 cm) were placed in 70% FA at 80 °C for 2 min. We removed the coverslips from the slides, and the slides were then incubated in 75%, 95%, and 100% ethanol (precooled at 20 °C) for 5 min. A total of 10 µL of hybridization solution, including 0.5 µL of the (AG_3_T_3_)_3_ probe, 1 µL of 5S rDNA, and 8.5 µL of the 2 × SSC, and 1 × TE mixture was dropped onto the chromosomes, and a cover glass (24 cm × 50 cm) was placed on top of the hybrid solution. The slides were then incubated at 37 °C for 2 h.

### 2.5. Image Capture and Analysis

The slides with hybridization were successively washed with 2 × SSC buffer and ddH_2_O. A total of 10 µL of 4,6-diamidino-2-phenylindole (DAPI) was dropped onto the air-dried chromosomes, then coverslips (24 cm × 50 cm) were placed on top. The slides were examined using an Olympus BX 63 fluorescence microscope combined with a Photometric SenSys Olympus DP70 CCD camera (Olympus Corporation, Tokyo, Japan). 

We conducted an analysis of the signal patterns by using the three best spreads. We used Photoshop version 2021 (Adobe Systems Inc., San Jose, CA, USA) to calculate the length of each chromosome, and each spread was measured three times, which obtained consistent chromosome data. The chromosomes were arranged by length from longest to shortest.

## 3. Results

### 3.1. Morphological Characteristics

*T. wallichiana* leaves were striped, more densely arranged into two irregular columns overlapping each other, thick in texture, and usually straight. Some were equal in width at the top or bottom or slightly narrow at the upper end and were symmetrical at the base. 

The leaves of *T.* × *media* were arranged in two irregular rows or slightly spirally. The leaves were striped, falcate curved, dense green, and slightly raised in the middle rib.

*T. yunnanensis*’ leaf texture was thin and soft, and the shape of the leaf was striped lanceolate.

*T. cuspidate* leaves were arranged in two irregular rows that were observably extending at an angle of about 45 degrees. Their leaves were bar-shaped, usually straight, thinly and slightly curved, narrow at the base, and had a short stripe. 

*T. wallichiana* var. *mairei* leaves were often wider and longer, the upper part was often narrow, the apex was acuminate, and the midvein belt was clearly visible. The leaves were yellowish green or green, and the green sideband was also wider and obvious. 

**Figure 1:**
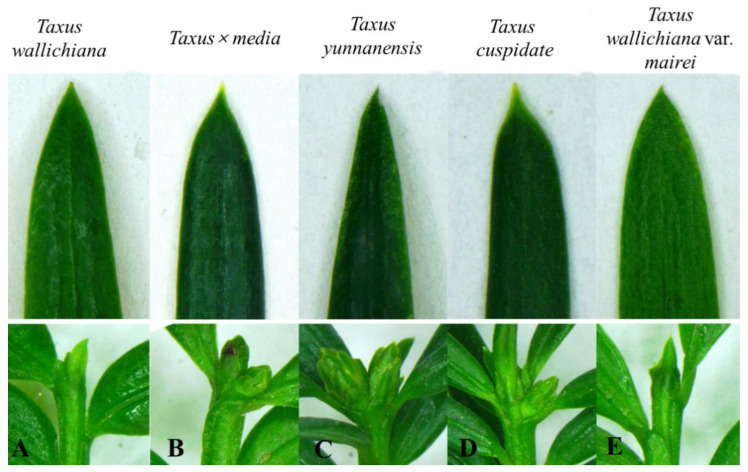
Morphological comparison of the leaves and bud. (**A**) *T. wallichiana,* (**B**) *T.* × *media*, (**C**) *T. yunnanensis*, (**D**) *T. cuspidate,* and (**E**) *T. wallichiana* var. *mairei*.

The leaf and bud morphology of these five plants are shown in Figure 1.

### 3.2. Chromosome 

**Figure 2:**
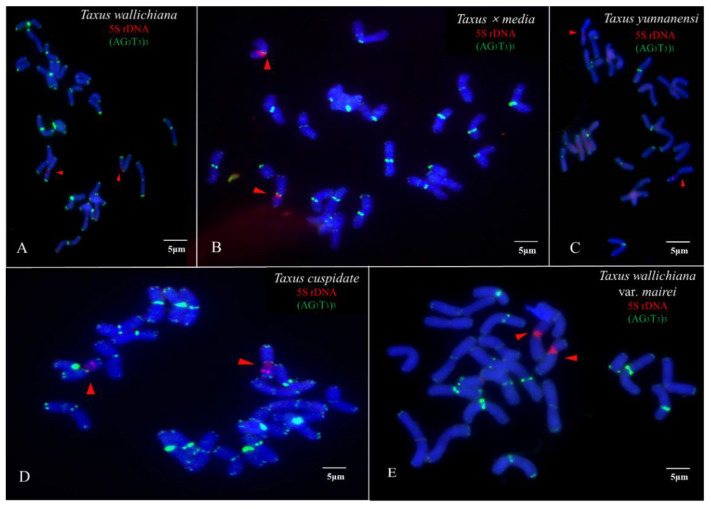
Visualization of the mitotic metaphase chromosomes of *T. wallichiana* (**A**), *T. × media* (**B**), *T. yunnanensis* (**C**), *T. cuspidate* (**D**), and *T. wallichiana* var. *mairei* (**E**) after fluorescence in situ hybridization (FISH). The first probe of (AG_3_T_3_)_3_ was labelled with 6-carboxyfluorescein (FAM) (green); the second probe of 5S rDNA was labelled with 6-carboxytetramethylrhodamine (TAMRA) (red). Chromosomes probed with 5′-TAMRA-labelled 5S rDNA (red fluorescence, arrow) are shown in (**A**–**E**). The concentration of all the probes was 10 µM. All the chromosomes were counterstained with 4,6-diamidino-2-phenylindole (DAPI) (blue). Scale bar = 5 µm.

**Figure 3:**
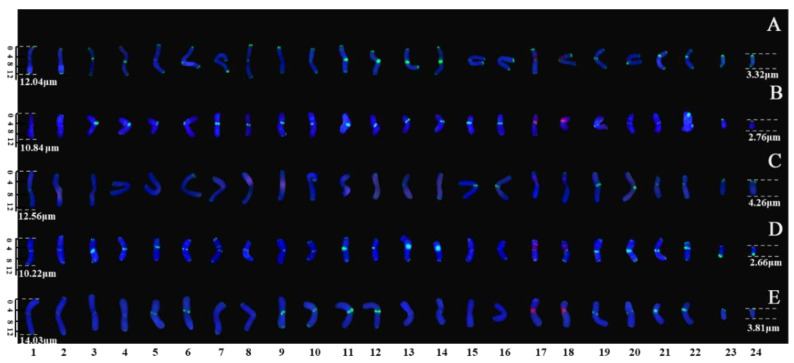
Mitotic chromosomes of five *Taxus* species rearranged from Figure 1. (**A**) *T. wallichiana*, (**B**) *T. × media,* (**C**) *T. yunnanensis*, (**D**) *T. cuspidate,* and (**E**) *T. wallichiana* var. *mairei.* Each chromosome is numbered according to its length, from the longest to the shortest chromosome. Scale bars range from 1 to 15 μm.

**Figure 4:**
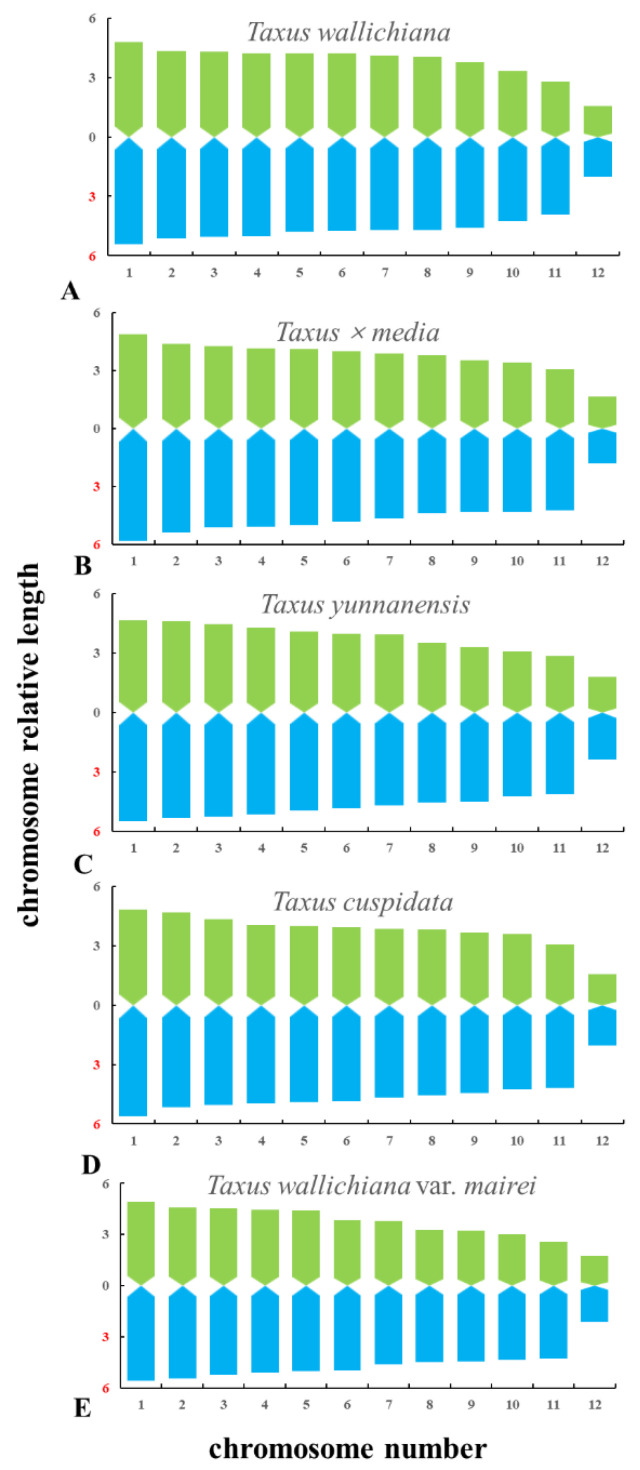
Karyotype idiograms of five *Taxus* species. (**A**) *T. wallichiana*, (**B**) *T. × media*, (**C**) *T. yunnanensis*, (**D**) *T. cuspidate,* and (**E**) *T. wallichiana* var. *mairei.* The x-axis indicates the chromosome number, whereas the y-axis indicates the relative chromosome length.

The metaphase and prometaphase chromosomes of five species of *Taxus* were analysed by FISH, as shown in Figure 2. The chromosome number of all five species of *Taxus* was 2n = 24. The individual chromosomes in Figure 2 were aligned based on length from the longest chromosome to the shortest chromosome, as shown in Figure 3. Except for the different karyotype formula of *T. wallichiana* var. *mairei*, the other four *Taxus* species have the same karyotype formula. The arm ratios of five *Taxus* species range from 2.6402 to 3.4198. The karyotype asymmetry index ranges from 54.33% to 55.56%. The karyotypes of the five *Taxus* species are shown in Table 2. The relative chromosome lengths of the five species of *Taxus* are shown in Figure 4.

### 3.3. Signal Distribution

**Figure 5:**
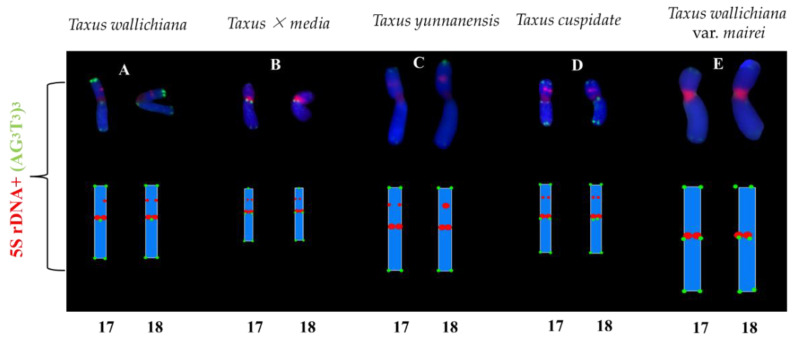
Signal distribution of the five *Taxus* species. (**A**) *T. wallichiana*, (**B**) *T. × media*, (**C**) *T. yunnanensis*, (**D**) *T. cuspidate,* and (**E**) *T. wallichiana* var. *mairei.* The idiograms were constructed based on the signal patterns of the chromosomes mentioned above and the chromosomes in Figure 1. The numbers at the bottom represent the number of chromosomes.

The (AG_3_T_3_)_3_ signal on chromosomes of five species of *Taxus* appeared at the centromere and proximal. The (AG_3_T_3_)_3_ signal showed slight differences, but it may help to count the chromosome number of the five species of *Taxus*. *T. cuspidate* and *T. × media* had two pairs of 5S rDNA signals, and the (AG_3_T_3_)_3_ signal location was shown to be in the centromere and chromosomal endpoint. The 5S rDNA signals of *T. wallichiana* and *T. yunnanensis* were distributed in centromeres and subtelomeres, and the number of 5S rDNA signals was consistent. The (AG_3_T_3_)_3_ signal of *T. wallichiana* and *T. yunnanensis* was distributed at the chromosome end. The 5S rDNA presented two signals at different locations in five species of *Taxus*, showing a great ability for chromosome discrimination. The 5S rDNA signals of *T. wallichiana* and *T. yunnanensis* were similar, but the individual 5S rDNA signal location of *T. yunnanensis* was more in the centre than in *T. wallichiana.* The 5S rDNA signals of *T. × media* and *T. cuspidate* were similar, but the (AG_3_T_3_)_3_ signals of *T. × media* in chromosomes 17 and 18 were fewer than in *T. cuspidate*, as shown in Figure 5.

## 4. Discussion

### 4.1. Karyotype 

*Taxus*, as a non-model gymnosperm, has been studied for organ transcriptome sequencing [18,42,43]. A draft genome assembly constructed by the whole genome sequencing of *T. baccata* has been published [19]. Although some molecular studies have been conducted on certain *Taxus* species [20,21], the whole genome sequencing of *Taxus* is still missing [22]. Although the complete genome sequencing of *Taxus* is missing, plant genomic diversity can also be reflected by a wide range of chromosome numbers [43]. Therefore, studying the chromosomes of five species of *Taxus* can also provide reference for genome research, to a certain extent.

Compared with angiosperms, gymnosperms are characterized by more consistent chromosome numbers and karyotypes [43]. Gymnosperms are one of the best studied plants in terms of chromosome number and karyotype [22,23,44,45]. To date, the *Taxus* family is the only gymnospermic family known to produce paclitaxel [46]. However, information on the *Taxus* family itself is limited, because the identification of the *Taxus* chromosomes is complicated due to their similar size and morphology [22]. 

In this study, chromosome numbers were the same as those previously published [47,48]. However, the karyotype formulas of the five species of *Taxus* were different from those previously published. In this study, except for the karyotype formula of *T. wallichiana* var. *mairei* being 2n = 2X = 22m + 2sm, the karyotype formulas of the other four *Taxus* are 2n = 2X = 24m. The previous study showed that the karyotype formulas of male *T. yunnanensis* were 2n = 24 = 24m, and those for females were 2n = 24 = 23m + 1sm [47] and 2n = 24 = 21m + 1sm + 2T [47]. The karyotype formula of *T.* × *media* was 2n = 24 = 20m + 2sm + 2st [48]. The karyotype formulas of *T. cuspidata* were 2n = 24 = 18m + 6sm [49], 2n = 24 = 18m + 4sm + 2T [48], and 2n = 24 = 4m + 6msm + 10m + 2sm + 2T [50]. The karyotype formula of *T. wallichiana* var. *mairei* was 2n = 24 = 20m + 2sm + 2T [47]. 

The karyotype formulas of different species are varied, which may be due to cell cycle synchronization and low chromosomal diffusion efficiency [22] or could be due to the differential accumulation of transposable factors [51,52]. The different treatment methods of *Taxus* root tip materials may also result in the inconsistency between the karyotype formula and previous studies.

### 4.2. Role of (AG_3_T_3_)_3_ and 5S rDNA 

By using the FISH technique, the (AG_3_T_3_)_3_ probe was used on five species of *Taxus*, and signals were found on all chromosomes, indicating that the chromosome shape of five species of *Taxus* was complete. Traditionally, (AG_3_T_3_)_3_ signals are usually detected at the chromosome end, but occasionally some signals are detected around and inside the centromere [31,53,54,55]. Studies have shown that the presence of non-telomere signals or interstitial telomere signals can indicate that the chromosome has undergone structural or quantitative rearrangement [56]. (AG_3_T_3_)_3_ signals were found around the centromeres in *T. wallichiana*, *T. × media*, *T. yunnanensis*, *T. cuspidata*, and *T. wallichiana* var. *mairei*, suggesting that these species may have undergone chromosome recombination. Studies have shown that there is more frequent gene loss and gain and genome-scale genomic rearrangement in gymnosperms [57]. The occurrence of this phenomenon may be related to the loss of reverse repeat sequences [1,58]. However, we did not lose sight of the fact that, due to the limited resolution of the FISH technique, no small gaps in the telomere signals due to chromosomal fusion were detected. 

The 5S rDNA is often used to identify differences between species and differences in the ploidy of the same species. It is also used to conduct species identification and the phylogenetic identification of extra-specific and inter-specific taxa [27,59,60]. The 5S rDNA signal may be located on every euchromosome [61], and may occur in the middle, near the middle, or at the end of the chromosome [24]. The 5S rDNA signal patterns were found at the centromeres of all five species of *Taxus*, but the 5S rDNA signal was also found at the proximal end of the chromosomes of *T. wallichiana*, *T. cuspidata,* and *T. yunnanensis*. Such results indicate that the 5S rDNA repeats are moving at each site, which may also be related to the deletion of inverted repeats (IR_S_). The signal patterns on the chromosomes of the five species of *Taxus* are different, which we believe may be because the plant adapts to the environmental disturbance and changes the gene expression profile to adapt to the environmental change [62], resulting in more gene deletion and acquisition. However, genomic studies are hampered by the lack of reference genomes and transcriptome of non-model plants, so the genomic level of gymnosperms is still being explored [63].

The signal patterns on the chromosomes of the five species of *Taxus* are different, which we believe may be because the plant adapts to environmental disturbances and changes the gene expression profile to adapt to the environmental change [62], resulting in more gene deletion and acquisition. However, genomic studies are hampered by the lack of reference genomes and the transcriptome of non-model plants, so the genomic level of gymnosperms is still being explored [63]. Of course, the more plausible reasons need to be further studied. The *Taxus* plants classification is mainly based on morphological description and geographical distribution [11], but some species of *Taxus* have little difference in phenotypic characteristics [9], which leads to the classification of species of *Taxus* being controversial. Chromosome data are useful for plant classification [64]. Hao et al. [1] inferred from chloroplast intergenic spacer and nuclear coding DNA that *T. wallichiana* var. *mairei* and *T. yunnanensis* are more closely related than *T. × media* [1]. Liu et al. [65] studied the phylogenetic relationship of *Taxus* from the perspective of Internal Transcribed Spacer (ITS), but excluded several controversial species such as *T. sumatrana* and *T. yunnanensis*. The 5S rDNA signals, which vary in location and intensity, were useful for intraspecific and interspecific taxonomic identification and phylogenetic relationship characterization [27]. 5S rDNA and (AG_3_T_3_)_3_ signals were shown to be located at different locations in five species in *Taxus*. Identification using only two signals might be unscientific, but it will contribute in providing a classification reference in *Taxus*.

There is a phylogenetic framework for variation in 5S rDNA distribution because taxa closeness is associated with a similarity in the FISH signal pattern [66,67]. The 5S rDNA signals were used as the main differentiators and (AG_3_T_3_)_3_ signals were auxiliary to determine the genetic relationship. The phylogenetic relationships between *T. cuspidate* and *T. × media* and between *T. wallichiana* and *T. yunnanensis* were the closest. On the other hand, the *T. wallichiana* var. *mairei* species was phylogenetically distant. The experimental results were similar to those of Wang et al. [68]. The discrepancy with previous results may be due to the different methods used. In addition, different origins and hybridization conditions may affect the relationships between these species. However, the use of 5S rDNA main signals and (AG_3_T_3_)_3_ auxiliary signals to determine the genetic relationship might be unscientific, so it should therefore be certified by more signal probes. In order to further study the phylogenetic relationships among the five *Taxus* species, molecular data need to be combined. 

The results of the oligonucleotide probes of five *Taxus* species were different, which may be helpful to draw oligonucleotide maps of *Taxus* plants in the future. If a specific oligonucleotide probe of *Taxus* is clear, it will be very convenient to identify *Taxus*. At the same time, we analysed the genetic relationship of five *Taxus* plants, which will be conducive to future cross breeding and use of heterosis.

## 5. Conclusions

Based on oligo-FISH, we found that the karyotype of the five *Taxus* species are different. This study provides chromosome characters that may be useful for karyotype comparisons with better pair identification within and between specific chromosome sets, as well as a physical map, contributing to the research on the cytogenetic composition of the *Taxus* genome.

## Figures and Tables

**Table 1:** Collection of seedling resources.

No.	Species	Location	Tissue
1	*Taxus wallichiana*	Xichang City, Sichuan Province, China	Seedling
2	*Taxus × media*	Ya’an City, Sichuan Province, China	Seedling
3	*Taxus yunnanensis*	Ya’an City, Sichuan Province, China	Seedling
4	*Taxus cuspidata*	Suqian City, Jiangsu Province, China	Seedling
5	*Taxus wallichiana* var. *mairei*	Ya’an City, Sichuan Province, China	Seedling

**Table 2:** Karyotype formulas of five species of *Taxus*.

Species	Karyotype	Karyotype Asymmetry Index	Arm Ratio
*T. wallichiana*	2n = 2x = 24m	55.56%	3.0087
*T. × media*	2n = 2x = 24m	55.09%	3.4198
*T. yunnanensis*	2n = 2x = 24m	55.56%	2.6402
*T. cuspidate*	2n = 2x = 24m	54.67%	3.0135
*T. wallichiana* var. *mairei*	2n = 2x = 22 m + 2sm	54.33%	2.8716

## Data Availability

All data and materials are included in the form of graphs in this article.
